# Plastic Operations

**Published:** 1845-11-01

**Authors:** F. H. Hamilton


### Plastic Operations.

*Continued from page* 66.

By F. H. Hamilton, M. D.

### PTOSIS AND ENTROPEON.

Mr. T. of Tyre, N. Y., aged 70. Has been afflicted many years with a Ptosis and Entropeon of both eyes. The Ptosis is evidently the result of partial paralysis of the levator palpebræ superioris, and the lids cannot be separated beyond the sixth of an inch. The Entropeon produces a constant opthalmia.

1st operation—Jan. 10, 1845—Removed a portion of the integument from the upper lid of left eye of an elliptical shape, one inch in length by three quarters of an inch in breadth, longest diameter parallel with the tarsal cartilages; closed the wound with four fine sutures.

2d operation—Jan. 15—Made the same operation on the opposite eye.

Both wounds united by adhesion and the eyelids are now sufficiently open and the entropeon with its consequences, has ceased.

I have seen to-day a girl upon whom I operated before the Medical class at Geneva, in the winter of 1844. The Ptosis affected only one eye and half concealed the cornea. No defect can now be seen in the lid.—I believe I am in the habit of removing more integument in these operations than most operators. If failures occur, it is generally because too little has been removed.

### LABIUM LEPORINUM.

E. Stafford, of Manchester, two years old—has a single cleft. Alveolar processes cleft also.

Operation Jan. 14th, 1845—1st, pared away the edges of the chasm and loosened up the adhesions from the gums &c. 2d, introduced three strong *interrupted* sutures, (harness makers silk) transfixing the *whole thickness* of the lip, at quite 3 lines from the margin of the wound; these I drew snug. 3d, applied 2 straps of adhesive plaster, broad upon the whole surface of the cheeks and forming two narrow tails which were made to interlace upon each other over the lip, and drawn together so firmly that the sutures were entirely relieved. 4th, laid a compress upon each cheek and over this a long roller to keep the straps firm in their places.

I have long since ceased the use of Hare-lip pins in these operations, and have reason to be greatly pleased with the change.

The advantages of this mode are that penetrating the whole thickness of the lip, the surfaces are held in more perfect coaptation,—most, if not all who use the hare-lip pin recommend that it shall penetrate two thirds only of the lip. There are no points of steel projecting and liable to be disturbed by a thousand accidents. Adhesive plasters can be used, and if employed as I have directed, they may be made to bear all the strain and thus avoid entirely the danger of the sutures tearing out; and especially do they guard against this at the time of the renewal of the dressings or the removal of the sutures, at which time, however violently the child may cry, (and cry he will) the edges are kept from separating by always having at least one leg of the adhesive straps, firm in its place.

The result has justified the practice in this case, as in every case in which I have adopted the above course. Of five operations made by me since 1839, not one has failed.

### PHYMOSIS AND STRICTURE.

Mr. ——— of Otisco, aged 30—Deaf and dumb. When a lad he received an injury from a kick upon his penis which produced at the time a violent inflammation. Since then a difficulty in passing his urine has always existed—within the last 3 or 4 months he can only pass a drop at a time. The prepuce is adherent over the whole extent of the glans, except about one quarter of an inch at the extremity.

Operation Jan. 11th, 1845—Dissected back the prepuce beyond the corona; attempted to introduce a fine silver lacrymal probe into the meatus, but it would not pass beyond half an inch; introduced a straight bistoury in the direction of the channel, one inch, when the point entered a large sinus, from which the urine escaped in a gush and with great force; whole of the glans completely cartilaginous; introduced a short, silver catheter, with a flat shoulder, and secured with tapes. No stricture beyond the first inch.

Wound healed kindly and patient is well.

### PHYMOSIS.

Medical student, aged 28—Has a congenital phymosis—not adherent. Cannot expose the glans.

Operation Feb. 1845—in presence of C. Hammond, medical student of Rochester. 1st, introduced three silk threads through the prepuce, transfixing it from side to side, behind the point at which the excision was to be made. 2d, drew forward the prepuce and excised one inch with scissors, then made an incision parallel to, and by the side of the fraenum, half an inch in length to enable the prepuce to retract more perfectly. 3d, cut the middle of each of the three threads where they crossed each other over the extremity of the glans, making six sutures, which were immediately tied and cut. 4th, simple dressing.

The peculiarity of this operation consists in introducing the sutures before the excision is made, and thus preventing that retraction of the outer integument which often renders it exceedingly difficult to pass the suture. I am indebted for the suggestion to M. Ricord.

This patient had a considerable hæmorrhage from the wound commencing a few hours after the operation and continuing several hours—arrested finally by applications of snow.

*Note.*—The operation of Blepheroplasty noticed in my former article as made upon Albert P. Heth, Jan. 8, 1845, and the patient dismissed upon the 18th, I have heard from by letter, dated August 9th. Dr. Heath, who has examined the case at my request, writes, "his eyes appear quite natural, and every thing is much improved; the tarsi do not turn out at all. He is very much pleased with the improvement, and indeed I th nk he ought to be," &c.

